# Sport dependent effects on the sensory control of balance during upright posture: a comparison between professional horseback riders, judokas and non-athletes

**DOI:** 10.3389/fnhum.2023.1213385

**Published:** 2023-07-31

**Authors:** Jean-Philippe Viseu, Eric Yiou, Pierre-Olivier Morin, Agnès Olivier

**Affiliations:** ^1^CIAMS, Université Paris-Saclay, Orsay, France; ^2^CIAMS, Université d’Orléans, Orléans, France; ^3^INSERM, CNRS, Institut de la Vision, Sorbonne Université, Paris, France; ^4^Institut Français du Cheval et de l’Equitation, Plateau technique de Saumur, Saumur, France

**Keywords:** sports expertise, balance control, sensory information, horseback riders, judokas

## Abstract

**Introduction:**

Compared to judokas (JU) and non-athletes (NA), horseback riders (HR) may develop specific changes in their sensory control of balance.

**Methods:**

Thirty-four international-level JU, twenty-seven international-level HR and twenty-one NA participated. Participants stood upright on a plateform (static condition) or on a seesaw device with an instability along the mediolateral (ML) or the anteroposterior (AP) direction (dynamic conditions). These conditions were carried out with eyes opened (EO) or closed (EC), and with (wF) or without a foam (nF). Experimental variables included conventional (linear), non-linear center-of-pressure (COP) parameters, Romberg Quotient (RQ) and Plantar Quotient (PQ).

**Results:**

Group effects. COP Surface (COPS) and standard deviation of COP along AP (SDY) were lower in HR than in JU in Static. SD Y was lower in HR than in JU in Dynamic AP. COP velocity (COPV) was lower in both HR and JU than in NA in Static and Dynamic. Sample entropy along AP and ML (SampEnY and SampEnX) were higher in HR than in JU in Static. SampEnY was higher in HR than in JU in Dynamic ML. Sensory effects. In EC, COPV was lower in JU than in NA in Dynamic AP, and lower in JU than in both HR and NA in Dynamic ML. In EO, COPV was lower in both JU and HR than in NA in Dynamic ML. RQ applied to COPS was lower in JU than in both HR and NA in Dynamic AP, and lower in JU than in HR in Dynamic ML. RQ applied to COPV was lower in JU than in both HR and NA in Static and Dynamic. PQ applied to COPS was higher in JU than in both HR and NA in Dynamic ML.

**Conclusion:**

Results showed that the effects of sport expertise on postural control could only be revealed with specific COP variables and were directionally oriented and sport-dependant. HR seem to rely more on vision than JU, thus revealing that the contribution of the sensory inputs to balance control is also sport-dependent. Results open up new knowledge on the specificity of sport practice on multisensory balance information during upright posture.

## 1. Introduction

Balance control is crucial to maintain our posture and perform our daily motor tasks efficiently. It also underlies the performance of highly complex motor skills such as those classically encountered in sports (Yiou et al., 2018). “Posture” can be defined as the relative position of body segments, and “balance” corresponds to a state where the external forces and moments acting on the body compensate each other (e.g., Bouisset and Do, 2008; Stone et al., 2015). Since the first study to date of standing posture on a mechanical platform (Hellebrandt, 1938), it is well-known that a perfect state of balance is never reached during postural maintenance, as evidenced by the existence of continuous center of pressure (COP) oscillations, referred to as “postural sway.” These COP oscillations do not exceed one-centimeter square under unconstrained bipedal conditions (Winter et al., 1997). It is generally assumed that under static conditions, the efficiency of the balance control system (i.e., the “postural performance”) is associated with the ability to minimize these COP oscillations (Paillard and Noé, 2015).

The effect of sport expertise on postural performance has been thoroughly investigated in the literature. As a general rule, experts have a better postural performance than non-athletes when tested under quiet standing posture, whether the sport involves static [e.g., pistol shooting (Aalto et al., 1990)] or dynamic balance control [e.g., golf (Sell et al., 2007), baseball (Butler et al., 2016), gymnastics (Asseman et al., 2008; Isableu et al., 2017; Busquets et al., 2018) or soccer (Paillard and Noé, 2006; Pau et al., 2015; Jadczak et al., 2019)]. The effect of sport expertise on postural performance is all the more important when the sport involves dynamic balance control on unstable support, as in surf (Paillard et al., 2011) and paddle board (Schram et al., 2016).

Sensory information used for postural control arises from the visual, proprioceptive, and vestibular systems (Peterka and Loughlin, 2004; Sozzi et al., 2011, 2019; Assländer and Peterka, 2014). Previous studies on sport expertise reported that the contribution of these sensory inputs to balance control changes with intensive training, with experts relying less on visual and vestibular inputs and more on somaesthetic inputs (Paillard, 2014, 2019). It is, however, noteworthy that, in the large majority of these studies investigating the effects of expertise on postural control, postural performance and sensory processing are typically investigated (i) through experimental conditions where the sensory information available are manipulated (e.g., by having participants standing with or without vision, on a hard or a compliant surface etc.), and (ii) in athletes practising a sport in the erect posture with the feet in contact with the ground (e.g., judo, soccer etc.). These two latter biomechanical characteristics of sports practice are common to the experimental conditions classically used for postural performance evaluation. It can thus be thought that a direct transfer of the postural skill of athletes to the experimental conditions has occurred. Now, in many other sports, athletes do not stand in an erect posture but are merely seated during their practice, and their feet are not in contact with the ground but with brackets, pedals etc. This is the case, for example, in horseback riding, canoeing, kayaking and rowing. Now, even for these sports, postural stability was reported to be improved in experts compared to non-athletes (Stambolieva et al., 2012 for canoeing and kayaking, Andreeva et al., 2021 for rowing), suggesting that sports practice has a positive effect on postural stability whatever the postural conditions of practice, seated or standing.

In line with the above mentioned studies (Stambolieva et al., 2012; Andreeva et al., 2021), Olivier et al. (2019) recently suggested that the postural skills developed by the horseback riders can be transferred to postural control under the quiet standing posture, as revealed with a higher postural performance in high-level horseback riders compared to non-athletes. It can, however, be questioned whether high-level horseback riding develops specific postural and/or sensory adaptations when compared to sports performed in a bipedal standing posture. To address this question, the postural performance and sensory processing of three different groups: high-level horseback riders, high-level judokas and non-athletes (control group), were compared during static and dynamic standing conditions. Judo was chosen as a reference model of bipedal sport because the postural behavior of judokas has been well described in the literature (Paillard, 2002; Barbado et al., 2016; Betelli et al., 2022; etc.) and has often been compared with other sports to reveal the specificities of expertise on postural control, e.g., with dance (Perrin et al., 2002), wrestling (Iwai et al., 2008), swimming (Itamar et al., 2013), and multisport [see (Hrysomallis, 2011) for a review]. The postural performance is expected to be better in the two high-level athletes groups than in the control group, and better in the judokas group than in the horseback riders group because of their intensive practice in the upright posture. It is also expected that athletes of both high-level groups rely less on visual inputs and more on somaesthetic inputs to control balance than the participants of the control group. However, because of their regular barefoot practice, judokas are expected to rely more on their somaesthetic inputs than horseback riders.

## 2. Materials and methods

### 2.1. Participants

Thirty-four international-level judokas (**JU** group), twenty-seven international-level horseback-riders (**HR** group) and twenty-one non-athletes (**NA** group) participated in the experiment (cf. Table 1 for anthropometrical characteristics). *A priori* calculation of the number of subjects required to obtain a statistical power of 0.80 and type 1 error of 0.05, showed that at least 20 subjects were needed (Paillard and Noé, 2006; Isableu et al., 2017; Olivier et al., 2019).

**TABLE 1:** Mean anthropometric characteristics of the three groups, and sport practice characteristics of the judokas and horseback riders groups.

	Horse riders (HR)	Judokas (JU)	Non-athletes (NA)
**Participants (*N*)**	**27**	**34**	**21**
Sex	♀ 9/♂ 18	♀ 18/♂ 16	♀ 12/♂ 9
Age (years)	33.63 ± 9.56	22.00 ± 3.89	22.10 ± 2.66
Body height (cm)	173.48 ± 6.86	172.76 ± 8.47	172.71 ± 8.58
Body mass (Kg)	66.33 ± 8.56	75.57 ± 14.15	66.87 ± 11.35
Years of practice	20.70 ± 8.77	16.15 ± 4.53	
Years of practice in competition	13.74 ± 7.61	8.36 ± 4.53	
Hours of practice per week	12.00 ± 0.21	13.00 ± 1.24	

Values are means ± one standard deviation.

Inclusion criteria were as follow: less than 3 h of sport per week in their leisure time at an amateur level for the NA group; more than 10 years of experience in competition at the national or international level and more than 5 h of practice per week for HR and JU group. Exclusion criteria were as follow (for all groups): lower limb length asymmetry, vestibular disorder, visual disorder, ankle injuries or neurological/musculoskeletal impairment in the past 3 months.

### 2.2. Apparatus and procedure

Each participant performed three experimental conditions (referred to as the “stability conditions”) where the level of stability differed: one static condition and two dynamic conditions. In the static condition, participants stood quietly upright and barefoot on a stabilometric platform (Medicapteurs^®^, Fusyo^®^ model) in a standardized posture with their heels 2 cm width-apart, arms by the sides of their body, and with a 30° external angle between their feet and the anteroposterior axis of the platform (Bizzo et al., 1985). The platform provided the 2D components of the COP. In the two dynamic conditions, participants stood in the same posture as in the static condition, but on a seesaw device (segment of cylinder, radius 55 cm and height 6 cm) located on the stabilometric platform. The seesaw device induced an instability along the mediolateral or the anteroposterior direction (Dynamic ML and AP conditions, respectively). In each of these three conditions of stability, the sensory inputs for postural control were manipulated (“sensory” conditions), i.e., participants were asked to stand upright with their eyes open for 31.6 s and then immediately continued with their eyes closed for another 31.6 s. In the same way, subjects stand upright with eyes opened (EO), eyes closed (EC), and with (wF) or without (noF) a foam (^®^Sidas Podiatech Expertene 220; height: 0.2 cm, hardness: 8 Shore, density: 220 kg.m^–3^) placed over the platform. All these experimental conditions (*n* = 12 in total) were randomized between participants to avoid rank effects. Each trial lasted 31.6 s. Previous studies reported that one single trial of this duration was sufficient to provide reliable values of the postural indicators used in the present study (Le Clair and Riach, 1996; Pinsault and Vuillerme, 2009; Baldini et al., 2013). Thirty seconds of rest was provided between conditions of stability.

The anteroposterior and mediolateral COP coordinates (X COP and Y COP) obtained with the platform were computed and sampled at 40 Hz, and filtered with a second-order Butterworth filter (8 Hz low-pass cut-off frequency). This sampling frequency has been shown to be sufficient to capture the COP dynamic changes during standing on a seesaw device (Guillou et al., 2007). The scripts for data processing were written in MATLAB R2018b (The Mathworks, Inc., Natick, MA, USA). The box plot figures were created using Minitab software, LLC, 2022.

### 2.3. Experimental variables

#### 2.3.1. Stabilometric evaluation

Postural control was investigated through the following stabilometric parameters (linear and non-linear) based on COP recordings.

##### 2.3.1.1. Linear parameters

– **The COP Surface** (in mm^2^) corresponds to the area of a 95% confidence ellipse. This parameter represents a measure of the COP spatial dispersion and postural performance (Geurts and Nienhuis, 1993; Asseman et al., 2004; Gagey and Weber, 2010; Ruhe et al., 2010; Paillard and Noé, 2015).
– The Standard Deviation of the COP (in mm) along the mediolateral (**SD X**) and anteroposterior (**SD Y**) axis of the platform, represents a measure of the variability of the COP trajectory (Geurts and Nienhuis, 1993; Gagey and Weber, 2010; Ruhe et al., 2010).
– The **Mean COP Velocity** (in mm.s^–1^) corresponds to the sum of the cumulated COP displacement divided by the total time of the trial. This parameter is a good and reliable indicator of stability (Geurts and Nienhuis, 1993; Gagey and Weber, 2010; Ruhe et al., 2010).

##### 2.3.1.2. Non-linear parameters

- The **Sample Entropy (SampEn).** The non-linearity of the postural sway dynamics was calculated by Sample Entropy based on positional coordinates COP time-series in the anterior-posterior (**SampEn Y**) and mediolateral position (**SampEn X**). Higher SampEn values indicate a lower predictability of future data points and a more significant irregularity within the time series. This has been interpreted as a reflecting a lower attentional investment in postural control (Stins et al., 2009; Isableu et al., 2017). SampEn was performed using the following input parameters for the analysis algorithms: 0.15 for tolerance (r) in proportion to the Standard Deviation of the signal and 2 for vector length (m). These values were selected based on the procedure suggested by different studies (Goldberger et al., 2000; Richman and Moorman, 2000; Yentes et al., 2013). Note that this procedure inspired studies on postural control with subjects of different sport expertise levels (Cavanaugh et al., 2007; Borg and Laxåback, 2010; Muelas Pérez et al., 2014; Janura et al., 2019). **SampEn** is largely independent of short record length and displays relative consistency under circumstances (Richman and Moorman, 2000).

##### 2.3.1.3. Quotients on COP parameters

- **The Romberg Quotient (RQ).** This variable reflects the influence of vision, or more precisely, the vision dependence on postural control (Lê and Kapoula, 2008; Hugentobler et al., 2016; Howcroft et al., 2017; Vartiainen et al., 2017; Zago et al., 2020). In all conditions of stability (static and dynamic), this variable was computed for each of the COP linear parameters described above as the ratio [COP variable in the **EO** condition/COP variable in the **EC** condition]. For example, for the COP surface, RQ was computed as follow:


$$\mathrm{RQ}=\left(\frac{\mathrm{COP}\,\mathrm{Surface}\,\mathrm{in}\,\mathrm{EC}\,\mathrm{condition}}{\mathrm{COP}\,\mathrm{Surface}\,\mathrm{in}\,\mathrm{EO}\,\mathrm{condition}}\right)*100$$

- **The Plantar Quotient (PQ).** This variable reflects the influence of plantar exteroceptive information on postural control (Foisy and Kapoula, 2016, 2017). For example, for the COP surface, PQ was computed as follow:


$$\mathrm{PQ}=\left(\frac{\mathrm{COP}\,\mathrm{Surface}\,\mathrm{in}\,\mathrm{EO}\,\mathrm{wF}\,\mathrm{condition}}{\mathrm{COP}\,\mathrm{Surface}\,\mathrm{in}\,\mathrm{EO}\,\mathrm{noF}\,\mathrm{condition}}\right)*100$$

Plantar Quotient provides information on the weight of plantar cutaneous afferents used in postural control (Foisy and Kapoula, 2016, 2017): the higher it is, the more the subject relies on the information arising from the feet to keep balance. Indeed, foam decreases the information arising from the feet, normally resulting in a decreased stability, indicated by a plantar quotient >100. In contrast, a plantar quotient <100 identifies a subject whose plantar cutaneous afferents impair postural control instead of being useful to balance, thus revealing a Plantar Exteroceptive Inefficiency (Foisy and Kapoula, 2016, 2017).

### 2.4. Statistical analysis

Mean values and standard deviations were calculated for each COP dependent variable. A Shapiro test and a Levene’s test were performed to ensure the normality of the data and the homogeneity of the variances, respectively. For each COP parameter, three separate Groups (JU vs. HR vs. NA) X two Vision conditions (EO vs. EC) X two Foam conditions (wF vs. nF) ANOVAs with repeated measures on the two last factors were conducted. In addition, differences between groups in terms of anthropometric characteristics, practice, RQ and PQ, were assessed with a one-way ANOVA. Due to the different sensory conditions tested, Bonferroni’s *post-hoc* test was used when required and the effect size on ANOVAs was estimated using partial eta squared (η2*p*). The statistical significance threshold was fixed at 0.05 for all analysis.

## 3. Results

The amount of practice in competition (in years) and the amount of weekly practice (in hours) were not significantly different between **HR** and **JU** (*p* = 0.73). In contrast, the amount of sports practice (in years) significantly differed between these two groups. **HR** has been practising longer than **JU** (*p* < 0.01). Also, there was a significant difference in age and weight (but not in height) between the three groups. **HR** were older than both **JU** and **NA** (*p* < 0.01), and **JU** weighted more than both **HR** and **NA** (*p* < 0.05).

### 3.1. Effects of the sensory conditions and the group on postural stability

**FIGURE 1:**
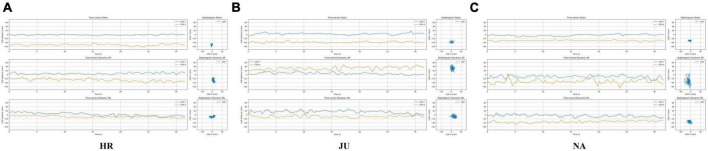
Examples of COP time-series and stabilograms in the three stability conditions with eyes open (one representative participant in each condition): static, dynamic AP and dynamic ML. **(A)** horseback rider (HR); **(B)** judoka (JU), and **(C)** non-athlete (NA) participants. Figures were created by Jean-Philippe Viseu.

Figure 1 shows typical COP time-series traces and stabilograms obtained in the three different stability conditions (i.e., static, dynamic AP and ML). As these traces were visually similar in all sensory conditions, only the traces in the EO condition were reported. COP parameters reported and analyzed below were extracted from these traces. For each stability condition taken separately, the ANOVAs showed a significant main effect of the Group on several stabilometric parameters (see Table 2 for statistics details).

**TABLE 2:** Main and interaction effects on the linear and non-linear COP parameters.

Postural parameters	Group			Vision			Foam			Vision × Group			Foam × Vision			Foam × Vision × Group		
	*F*_(2,79)_	*P*	η ^2^_p_	*F*_(2,79)_	*p*	η ^2^_p_	*F*_(2,79)_	*p*	η ^2^_p_	*F*_(2,79)_	*p*	η ^2^_p_	*F*_(2,79)_	*p*	η ^2^_p_	*F*_(2,79)_	*p*	η ^2^_p_
**Stability condition:**																		
**Linear parameters**																		
**COP surface**																		
Static	**5.82**	**<0.01**	**0.86**	**46.75**	**<0.001**	**1.00**	1.56	0.22		1.56	0.22		0.33	0.56		0.65	0.53	
Dynamic AP	1.24	0.30		**475.86**	**<0.001**	**1.00**	**14.91**	**<0.001**	**1.00**	**14.91**	**<0.001**	**1.00**	2.53	0.12		1.06	0.35	
Dynamic ML	0.72	0.49		**512.74**	**<0.001**	**1.00**	**7.63**	**0.001**	**0.94**	**7.63**	**0.001**	**0.94**	0.01	0.91		1.71	0.19	
**Mean COP velocity**																		
Static	**184.79**	**<0.001**	**1.00**	**181.43**	**<0.001**	**1.00**	**8.18**	**<0.001**	**0.95**	**8.18**	**<0.001**	**0.95**	0.31	0.58		0.16	0.86	
Dynamic AP	**6.35**	**<0.01**	**0.89**	**853.16**	**<0.001**	**1.00**	**24.04**	**<0.001**	**0.95**	**24.04**	**<0.001**	**0.95**	0.46	0.49		1.44	0.24	
Dynamic ML	**12.71**	**<0.001**	**1.00**	**769.24**	**<0.001**	**1.00**	**15.32**	**<0.001**	**1.00**	**15.32**	**<0.001**	**1.00**	0.10	0.75		1.37	0.26	
**SD X**																		
Static	2.71	0.07		**12.40**	**<0.001**	**0.94**	1.02	0.36		1.02	0.36		1.34	0.25		1.17	0.31	
Dynamic AP	0.23	0.79		**283.20**	**<0.001**	**1.00**	**8.22**	**<0.001**	**0.95**	**8.22**	**<0.001**	**0.95**	**5.17**	**0.03**	**0.61**	1.04	0.36	
Dynamic ML	0.29	0.75		**537.68**	**<0.001**	**1.00**	1.92	0.15		1.92	0.15		0.21	0.65		2.80	0.07	
**SD Y**																		
Static	**6.88**	**<0.01**	**0.91**	**43.44**	**<0.001**	**1.00**	1.78	0.18		1.78	0.18		0.31	0.58		0.12	0.89	
Dynamic AP	**8.20**	**<0.001**	**0.95**	**435.89**	**<0.001**	**1.00**	**15.99**	**<0.001**	**1.00**	**15.99**	**<0.001**	**1.00**	0.01	0.95		0.32	0.73	
Dynamic ML	1.18	0.31		**238.32**	**<0.001**	**1.00**	**12.84**	**<0.001**	**1.00**	**12.84**	**<0.001**	**1.00**	0.57	0.45		0.26	0.77	
**Non-linear parameters**																		
**SampEn X**																		
Static	**4.40**	**0.02**	**0.75**	0.82	0.37		0.44	0.51		1.21	0.30		0.12	0.73		2.12	0.13	
Dynamic AP	1.02	0.13		0.76	0.39		1.90	0.17		2.82	0.07		1.61	0.21		2.54	0.09	
Dynamic ML	0.24	0.20		0.08	0.78		**7.94**	**0.01**	**0.79**	0.04	0.96		2.30	0.13		1.14	0.32	
**SampEn Y**																		
Static	**3.61**	**0.03**	**0.65**	**4.15**	**0.04**	**0.52**	0.80	0.37		2.31	0.11		2.10	0.15		2.54	0.09	
Dynamic AP	1.38	0.26		1.23	0.27		0.37	0.54		1.61	0.21		1.04	0.31		1.89	0.16	
Dynamic ML	**3.52**	**0.03**	**0.64**	**4.84**	**0.03**	**0.58**	**5.14**	**0.03**	**0.61**	2.35	0.10		1.43	0.24		1.33	0.27	

COP surface (in mm^2^): area of the 95% confidence of the COP ellipse; Mean COP Velocity (in mm.s^–1^): sum of the cumulated COP displacement divided by the total time of acquisition; SD X, SD Y: standard deviation of the COP length along the mediolateral and anteroposterior axis, respectively; SampEn X, SampEn Y: sample entropy measures along the mediolateral and anteroposterior axis, respectively. In bold: significant effect on group and interaction effects. In shaded: significant *post hoc* effect in one given stability condition.

The ANOVAs showed a significant main effect of the Vision on all COP parameters and in all conditions of stability with higher values in EC condition than in EO condition.

The ANOVAs showed a significant main effect of the Foam on several stabilometric parameters and conditions of stability with higher values in **wF** condition than in **noF.**

#### 3.1.1. Static condition

##### 3.1.1.1. COP surface

The ANOVAs showed a significant main effect of the Group (*p* < 0.01). *Post-hoc* tests showed that this variable was lower in HR (163.79 ± 106.97 mm^2^) than in JU (245.21 ± 123.82 mm^2^).

##### 3.1.1.2. Mean COP velocity

The ANOVAs further revealed a significant main effect of the Group (*p* < 0.001). *Post-hoc* tests showed that this variable was lower in both HR (11.42 ± 3.63 mm.s^–1^) and JU (11.58 ± 3.70 mm.s^–1^) than in NA (46.46 ± 14.73 mm.s^–1^). The ANOVAs further showed a significant **Vision × Group** interaction effect [*F*_(2, 79)_ = 8.18, *p* = 0.001, η^2^*p* = 0.95]. *Post-hoc* tests, however, did not show any significant effect.

##### 3.1.1.3. SD Y

There was a significant main effect of the Group on SD Y (*p* < 0.01). *Post-hoc* tests showed that SD Y was lower in HR (3.81 ± 1.12 mm) than in JU (4.91 ± 1.24 mm) in the Static condition.

##### 3.1.1.4. SampEn X

The ANOVAs showed a significant main effect of the Group (*p* = 0.02). *Post-hoc* tests showed that **SampEn X** was higher in **HR** (2.39 ± 0.04) than in **JU** (2.36 ± 0.05).

##### 3.1.1.5. SampEn Y

The ANOVAs showed also a significant main effect of the Group (*p* = 0.03). *Post-hoc* tests showed that **SampEn Y** was higher in **HR** (2.39 ± 0.05) than in **JU** (2.35 ± 0.04) in this condition.

#### 3.1.2. Dynamic AP condition

##### 3.1.2.1. COP surface

The ANOVAs showed a significant **Vision × Group** interaction effect [*F*_(2,79)_ = 14.91, *p* < 0.001, η^2^*p* = 1]. *Post-hoc* tests, however, did not show any significant effect.

##### 3.1.2.2. Mean COP velocity

The ANOVAs showed a significant main effect of the Group (*p* < 0.01). *Post-hoc* tests showed that this variable was lower in both **HR** (33.16 ± 10.94 mm.s^–1^) and **JU** (30.37 ± 9.32 mm.s^–1^) than in **NA** (40.09 ± 17.93 mm.s^–1^). The ANOVAs further showed a significant **Vision × Group** interaction effect [*F*_(2,79)_ = 24.04, *p* < 0.001, η^2^*p* = 1]. In the **EC** condition, *Post-hoc* tests showed that this parameter was lower in **JU** (38.33 ± 11.03 mm.s^–1^) than in **NA** (50.32 ± 23.63 mm.s^–1^).

##### 3.1.2.3. SD X

The ANOVAs showed a significant **Vision × Group** interaction [*F*_(2,79)_ = 8.22, *p* = 0.001, η^2^*p* = 0.95]. *Post-hoc* tests, however, did not show any significant effect. The ANOVAs showed a significant **Foam × Vision** interaction [*F*_(2,79)_ = 5.17, *p* < 0.05, η^2^*p* = 0.61]. *Post-hoc* tests, however, did not show any significant effect.

##### 3.1.2.4. SD Y

The ANOVAs showed a significant main effect of the Group (*p* < 0.001). *Post-hoc* tests showed that this variable was also lower in HR (10 ± 2.09 mm) than in **JU** (11.24 ± 2.12 mm). The ANOVAs showed a significant **Vision × Group** interaction [*F*_(2,79)_ = 15.99, *p* < 0.001, η^2^*p* = 1]. *Post-hoc* tests, however, did not show any significant effect.

#### 3.1.3. Dynamic ML condition

##### 3.1.3.1. COP surface

The ANOVAs showed a significant **Vision × Group** interaction effect [*F*_(2,79)_ = 7.93, *p* = 0.001, η^2^*p* = 0.94]. *Post-hoc* tests, however, did not show any significant effect.

##### 3.1.3.2. Mean COP velocity

The ANOVAs showed a significant main effect of the Group (*p* < 0.001). Once again, *post-hoc* tests showed that this variable was lower in both **HR** (36.35 ± 13.64 mm.s^–1^) and **JU** (30.38 ± 10.59 mm.s^–1^) than in **NA** (45.03 ± 14.24 mm.s^–1^).

The ANOVAs further showed a significant **Vision × Group** interaction effect [*F*_(2,79)_ = 15.62, *p* < 0.001, η^2^*p* = 1] condition. In the **EO** condition, *post-hoc* tests showed that this parameter was lower in both **JU** (21.98 ± 7.37 mm.s^–1^) and **HR** (20.28 ± 6.13 mm.s^–1^) than in **NA** (38.64 ± 14.87 mm.s^–1^) group (with *p* < 0.01 and *p* < 0.001, respectively). In the **EC** condition, *post-hoc* tests also showed that it was lower in **JU** (38.77 ± 13.81 mm.s^–1^) than in both **HR** (52.41 ± 21.14 mm.s^–1^) and **NA** (51.43 ± 13.61 mm.s^–1^) in the **Dynamic ML** condition (with *p* < 0.01 and *p* < 0.01, respectively).

##### 3.1.3.3. SD Y

The ANOVAs showed a significant **Vision × Group** interaction [*F*_(2,79)_ = 12.84, *p* < 0.001, η^2^*p* = 1]. *Post-hoc* tests, however, did not show any significant effect.

##### 3.1.3.4. SampEn Y

The ANOVAs showed a significant main effect of the Group (*p* = 0.03). *Post-hoc* tests showed that this variable was higher in **HR** (2.41 ± 0.03) than in **JU** (2.38 ± 0.03) in the **Dynamic ML** condition.

**FIGURE 2:**
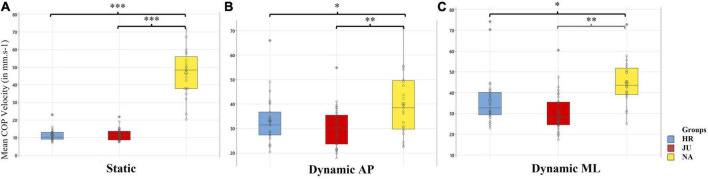
Between groups comparison of Mean COP Velocity (in mm.s^−1^) in the static, **(A)**, dynamic AP **(B)**, and dynamic ML **(C)** conditions. Reported are boxplot representations of each group, horseback riders (HR), judokas (JU), and non-athletes (NA), with individual data points (circles). ^*^, ^**^, ^***^Indicate a significant difference between two groups as revealed with the *post-hoc* tests, with *p* < 0.05, *p* < 0.01, and *p* < 0.001, respectively.

Group effects and the *post-hoc* tests on **Mean COP Velocity** are reported in Figure 2 for the three conditions of stability.

### 3.2. Effect of the group on Romberg and plantar quotients in the different stability conditions

#### 3.2.1. Romberg quotients (RQ)

##### 3.2.1.1. RQ applied to the COP surface

There was a significant main effect of the Group on this variable in both the **Dynamic AP** and **ML** conditions (but not in the **static** condition). More specifically, *post-hoc* tests showed that this parameter was lower in **JU** (304.16 ± 256.49) than in both **HR** (615.70 ± 382.34) and **NA** (484.66 ± 292.17) in the **Dynamic AP** condition [*F*_(2,79)_ = 14.57, *p* < 0.001, η^2^*p* = 1]. It was also lower in **JU** (370.61 ± 218.79) than in **HR** (560.93 ± 423.75) in the **Dynamic ML** condition [*F*_(2,79)_ = 7.37, *p* < 0.001, η^2^*p* = 1].

##### 3.2.1.2. RQ applied to the COP mean velocity

The ANOVAs showed a significant main effect of the Group on this variable in all conditions of stability. More specifically, *post-hoc* tests showed that this variable was lower in **JU** (119.33 ± 28.06) than in both **HR** (140.97 ± 31.88) and **NA** (147.41 ± 46.93) in the **Static** condition [*F*_(2,79)_ = 7.62, *p* = 0.001, η^2^*p* = 0.94]. Similarly, it was lower in **JU** (184.91 ± 50.15) than in both **HR** (283.10 ± 87.80) and **NA** (244.96 ± 67.34) in the **Dynamic AP** condition [*F*_(2,79)_ = 24.21, *p* < 0.001, η^2^*p* = 1]. Finally, this variable was lower in **JU** (194.72 ± 55.75) than in both **HR** (270.36 ± 88.79) and **NA** (218.33 ± 52.25) in the **Dynamic ML** condition [*F*_(2,79)_ = 15.35, *p* < 0.001, η^2^*p* = 1].

##### 3.2.1.3. RQ applied to the SD X and Y, SampEn X and Y

The ANOVAs did not show any significant effect of the Group.

**FIGURE 3:**
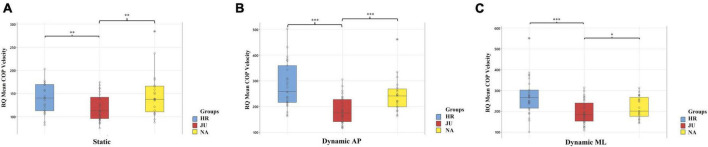
Between groups comparison of the Romberg quotients (RQ) applied to the mean COP velocity in the static **(A)**, dynamic AP **(B)**, and dynamic ML **(C)** conditions. Reported are boxplot representations of each group, horseback riders (HR), judokas (JU), and non-athletes (NA), with individual data points (circles) of COP parameter values. ^*^, ^**^, ^***^Indicate a significant difference between two groups as revealed with the *post-hoc* tests, with *p* < 0.05, *p* < 0.01, and *p* < 0.001, respectively.

Significant *post-hoc* tests on **RQ** on **Mean COP Velocity** are reported for illustration in Figure 3 for the three conditions of stability.

#### 3.2.2. Plantar quotients (PQ)

##### 3.2.2.1. PQ applied to the COP surface

**FIGURE 4:**
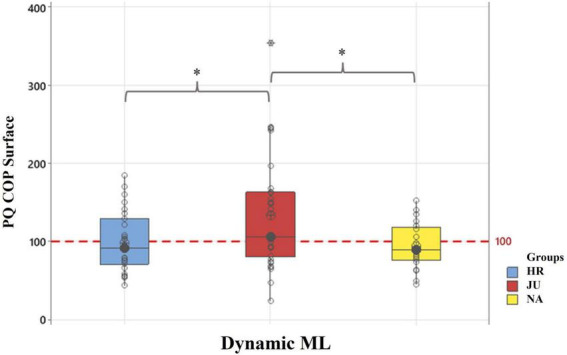
Plantar quotients (PQ) applied to the COP surface parameter in dynamic ML condition. reported are boxplot representations of each group, horseback riders (HR), judokas (JU), and non-athletes (NA), with individual data points (circles) of COP Surface parameter values. *Indicates a significant difference between two groups as revealed with the *post-hoc* tests, with *p* < 0.05.

The ANOVAs showed a significant main effect of the Group on this variable in the **Dynamic ML** condition [*F*_(2,79)_ = 4.99, *p* < 0.01, η^2^*p* = 0.79]. More specifically, *post-hoc* tests showed that this variable was higher in **JU** (100.13 ± 17.79) than in both **HR** (94.35 ± 24.65, *p* = 0.03) and **NA** (86.69 ± 16.27, *p* = 0.02). Note that this variable reached a mean value above 100 in **JU** while it reached a mean value below 100 in **HR** and **NA**. See details of the *post-hoc* tests in Figure 4.

##### 3.2.2.2. PQ applied to the mean COP velocity, SD X and Y, SampEn X and Y

The ANOVAs did not show any significant effect of the Group.

## 4. Discussion

Results of the present study showed that the intensive practice of horseback riding and judo improved balancing abilities in the upright posture. This effect was expected according to the literature on the effects of sport expertise on balance control (e.g., Paillard, 2014, 2019). More originally, results further showed that the effects of sports practice could only be revealed with specific COP variables. In addition, these effects were directionally oriented. Finally, results showed that HR had better stability and relied more on vision than JU to maintain balance, thus revealing that the contribution of the sensory inputs to balance control is sport-dependent.

### 4.1. Effects of sport expertise and sport specificity on postural performance

#### 4.1.1. Effects of sport expertise

In the present study, postural performance was investigated through conventional (linear) COP parameters (mean COP velocity, COP surface, SD Y and SD X) and non-linear COP parameters (SampEn X and SampEn Y). It is generally assumed that the former are indicators of postural stability (Paillard and Noé, 2015; Quijoux et al., 2021) while the latter are indicators of the amount of attentional investment in postural control (Stins et al., 2009; Yentes et al., 2013; Isableu et al., 2017; Montesinos et al., 2018; Thalassinos et al., 2018). More specifically, a more irregular COP trajectory, as assessed by higher SampEn values, has been suggested to be associated with more automaticity and has been proposed to reflect a reduction of the amount of attention invested in the control of posture.

Results showed that the mean COP velocity was the sole among the linear COP parameters that could differentiate high-level athletes from NA. More specifically, this parameter was lower in both JU and HR than in NA, regardless of the stability condition (i.e., static or dynamic) and the direction of the instability induced by the seesaw (i.e., ML or AP). It thus seems that the beneficial effects of sport expertise on postural stability do not depend on the postural conditions of practice, upright or seated. This statement is in line with current evidence from the literature suggesting that practising any kind of sport at a high-level has a beneficial effect on postural stability (Kiers et al., 2013; Olivier et al., 2019; Paillard, 2019). Our findings further suggest that the mean COP velocity is the most sensitive parameter to differentiate high-level athletes from NA.

In contrast to the mean COP velocity, the COP surface area, SD X and SD Y were not sensitive to the effect of sport expertise. Two of these parameters (the COP surface and SD Y) were, however, sensitive to the sport practised by the athletes (sport specificity), i.e., horseback riding or judo. The present results thus suggest that each of the linear COP parameters is merely sensitive to a specific aspect of sport practice, i.e., the level of sport expertise or the sport practiced.

The analysis of non-linear COP parameters (SampEn X and SampEn Y) completed this picture of the effects of sport expertise on postural control. In contrast to the linear COP parameters, results showed that none of the non-linear parameters could differentiate the two groups of high-level athletes from NA, in both static and dynamic conditions. This finding thus suggests that the level of sport expertise did not influence the amount of attentional investment in postural control, at least for the experimental tasks of the present study. This finding markedly contrasts with the study of Isableu et al. (2017) that compared postural control of expert gymnasts (G) to that of non-gymnasts (NG) during bipedal closed-eyes quiet standing using conventional and non-linear COP parameters. These authors found that only non-linear parameters could differentiate G from NG, with G having a higher entropy values than NG. The authors concluded that “a finer-grained analysis of the dynamic patterns of the COP displacements is required to uncover an effect of gymnastic expertise on postural control in non-demanding postural stance,” and that “less attentional investment, i.e., a more fully automatized form of balance, is required in experts in sports requiring fine postural control than controls.” This discrepancy with the results of the present study might be ascribed to a specific effect of the sport practiced by the athletes, i.e., gymnastic, judo or horseback riding.

Globally taken, results of the present study suggest that the two high-level groups had a better postural stability than NA (as revealed with the linear parameters), which was achieved with the same level of complexity of COP time-series. This finding suggests that the amount of attention invested in postural control was similar in these two groups. In the study of Isableu et al. (2017), G had the same level of postural stability than NG, which was achieved with a lower amount of attentional investment. In other words, considering the ratio between postural stability and the amount of attentional investment, these findings suggest that sports experts developed optimal postural control, regardless of the sport practised in the present study and the study of Isableu et al. (2017).

#### 4.1.2. Effects of sport specificity

Results further showed that the COP surface area and SD Y (but not SD X) were both lower in HR than in JU, which shows that HR had a better stability than JU. As stated in the introduction, a difference of stability was expected between these two groups, but the “direction” of this difference was not. Indeed, better stability was, in fact, merely expected in JU than in HR because, in contrast to horseback riding, judo involves intensive practice in the standing posture, which corresponds to the posture where stability was evaluated in the present study. In high-level athletes, postural stability performance under laboratory experimental conditions cannot be predicted based on the similitude with the postural condition of intensive practice, seated or standing.

Now, it is noteworthy that balance control is particularly crucial in horseback riding since any instability caused by the (sometimes) unpredictable movements of the horse is potentially a source of fall in height, with the risk of serious injuries (Silver, 2002; Pugh and Bolin, 2004; Lewis et al., 2018). In addition, the cutaneous surface of the lower body that is solicited during sports practice is more extended in horseback riding than in judo. In horseback riding, this surface includes the feet, the inner part of the legs and the buttock (in contact with the stirrups and the saddle, respectively) while in judo, it solely includes the feet (in contact with the support surface). An increased solicitation of the cutaneous receptors of the lower body during horseback practice may have contributed to developing an increased sense of body position and movement (i.e., an increased proprioception) in HR, with a consequent better balance control than in JU. In congruence with this hypothesis, the experimental stimulation of lower legs cutaneous receptors via the wear of garments has been shown to improve balance control on a wobble board in high-level athletes (handball players), especially those with best balance abilities at baseline (Baige et al., 2020). Finally, the instability on the seesaw elicited in the present experiment and the instability on the stirrups elicited during horseback riding requires somehow similar balance control skills. Because of this similitude, a transfer of balance skills from practice to the experiment may have occurred to a greater extent in HR compared to JU [for a review on the relationship between sport expertise and postural skills, see Paillard (2019)]. The results that HR performed better than JU especially along the AP direction (but not the ML direction), as attested by their lower SD Y value in the Dynamic AP condition (and also in the static condition), are congruent with this statement. Indeed, during horseback riding, balance is mainly challenged in the AP direction due to the intermittent horse accelerations, brakes, jumps etc., which are merely forwardly oriented rather than laterally oriented. A similar directional effect of horseback riding on balance control was reported when instability was experimentally elicited on a horse or on a simulator (Olivier et al., 2017, 2019). Such directional effect of expertise in sport on balance control in the standing posture has also been reported in the study by Glass and Ross (2021), which included 167 high level athletes split in four different groups (basketball, football, tennis, and cross-country). Globally taken, these findings suggest that attention should be paid to the profiling of COP direction to improve the understanding of balance behavior related to sport-specific sensorimotor adaptations.

The analysis of the non-linear COP parameters completed the picture of the effects of sport specificity on postural control. As for the linear COP parameters, results showed that non-linear parameters could differentiate JU from HR. More specifically, SampEN X was higher in HR than in JU in the static condition, and SampEN Y was higher in HR group than in JU in both the static and dynamic ML conditions. Altogether, these results suggest that HR has better postural stability than JU (as argued in the paragraph above), achieved with an even lower amount of attentional investment in postural control. This optimal postural control of HR is particularly significant if one considers postural control along the anteroposterior (Y) direction, since SDY and sampEN Y were revealed to be lower and higher than JU, respectively, in the both the static and dynamic conditions. This finding thus further reinforces the directional effect of sport expertise on postural control, which seems to be a sport practice-dependant effect.

### 4.2. Multisensory reweighting effects on COP parameters

#### 4.2.1. Vision effects

The Romberg Quotients (RQ) applied to the COP Surface and to the Mean Velocity were globally higher in both HR and NA than in JU, with no difference between HR and NA. JU therefore, seems to rely less on visual inputs to control balance than HR and NA. Our study confirms the hypothesis of reweighting of visual information toward proprioceptive information acquired with sports training, but only for a single group of high-level athletes (Paillard, 2014, 2019). A recent study (Olivier et al., 2019) found that HR had a lower visual dependence than NA, as revealed with the lower RQ applied to the same stabilometric parameter (specifically in the dynamic AP condition). In the present study, HR were indeed older than in precedent study (Olivier et al., 2019), a factor that has been showed to increase the Romberg Quotients (Lê and Kapoula, 2008; Rugelj et al., 2015) and may explain the lack of significant difference between HR and NA.

#### 4.2.2. Plantar effects

Our results showed that the Plantar Coefficient (PQ) applied to the COP surface was below 100 for both HR and NA, whereas it was above 100 for JU. These results suggest that JU was less stable on foam than on hard surface, whereas HR and NA were less stable on hard surface than on foam. Our results further suggest that HR and NA were less dependent on plantar information than JU (Foisy and Kapoula, 2016). Interestingly, Foisy and Kapoula showed that subjects with PQ values below 100 had a different multisensory visual-podal reweighting than subjects with PQ values above 100. The former had a less efficient oculomotricity and a lower sensitivity to plantar stimulations than the latter (Foisy et al., 2015). Our results suggest that the weight of plantar information is lower than the weight of visual information for postural control in HR compared to NA. Foisy and Kapoula (2017) suggested that this relative visual and plantar dependence can also be attributed to a latent somatosensory dysfunction on plantar information. Plantar irritant stimulus (PIS) is considered to be a noise that prevents the CNS from properly processing and using the somatosensory afferents of the feet for the control of balance and ocular vergence (Assländer and Peterka, 2014; Foisy and Kapoula, 2017; Peterka et al., 2018). This result is congruent with the results obtained with the RQ described in the above paragraph and suggest that the visual dependence of the HR and NA might be at the detriment of other sensory information (i.e., plantar information).

### 4.3. Limitations

This study has at least two limitations. First, performing only one trial in each testing condition might be seen as a methodological issue as one may criticize that one trial is not sufficient to ensure capturing the representative COP performance. We chose this method for the following reasons: previous studies showed that one single trial was sufficient to provide reliable values of the COP parameters used in the present study (e.g., Le Clair and Riach, 1996, Pinsault and Vuillerme, 2009); this method had previously been applied in the literature to investigate the effects of sport expertise and sport specificity on postural control under different stability and sensory conditions (e.g., Paillard, 2017, 2019 for reviews); given the relatively high number of participants included in each group (which implies that a large number of trials was performed in each condition), and the high effects sizes reported for most variables with a significant difference across groups/conditions, we feel confident on the reliability of our data; Finally, Now, participants of the present study underwent many experimental conditions. Therefore, to avoid the effect of fatigue, we chose to have them perform one single trial in each condition, rather than three as it can be done in the literature. Second, we have interpreted the between-group difference in the SampEn values as reflecting a difference in the attentional investment in postural control. This interpretation should be taken with caution as it needs to be confirmed with additional experiments involving traditional dual-task paradigm [e.g., (Vuillerme and Nougier, 2004)].

## 5. Conclusion

Very few studies have to date investigated the sensory control of balance in high-level athletes for whom the upright standing posture is not the ecological condition of practice. Results of the present study showed that intensive practice of horseback riding and judo improved balancing abilities in the upright posture and that HR had better stability than JU. However, these effects of sports practice could only be revealed with specific COP variables, and they were directionally oriented. In addition, HR relies more on vision than JU to maintain balance, thus revealing that the contribution of the sensory inputs to balance control is sport-dependent. These results reveal new knowledge on the specificity of sports practice on the sensory control of balance during upright posture. They may also be helpful for selecting relevant variables related to postural control for the follow-up of athletes, e.g., in prophylactic training, rehabilitation or optimization of horse-rider coupling.

## Data availability statement

The raw data supporting the conclusions of this article will be made available by the authors, without undue reservation.

## Ethics statement

This research did not receive any specific grant from funding agencies in the public, commercial, or not-for-profit sectors. All participants gave written informed consent after being instructed about the nature and purpose of the experiment, which was approved by the Ethics Committee of University Paris-Saclay (CER-Paris-Saclay-2021-049). This study complied with the standards established by the Declaration of Helsinki. The patients/participants provided their written informed consent to participate in this study.

## Author contributions

J-PV and AO designed and performed the experiments. J-PV and P-OM processed and analyzed data. J-PV contributed reagents, materials, and analysis tools and prepared figures and tables. J-PV, EY, and AO wrote the manuscript. EY and AO reviewed drafts of the manuscript. All authors signed the final approval for publication.
